# The Death-Rate of England
*An Inquiry into the different Proportions of Death produced by certain Diseases in different Districts in England. By Edward Headlam Greenhow, M.D. With an Introductory Report on the Preventibility of Certain Kinds of Premature Death. By John Simon, F.R.S. Blue Book. London: 1858.


**Published:** 1858-10

**Authors:** 


### SANITARY REVIEW.

OCTOBER 1858.

### THE DEATH-RATE OF ENGLAND.*

"Pallida mors æquo pulsat pede pauperum tabernas
Regumque turres."

So sang the poet Horace, in the Augustan age of Rome; and it is only of late years that the world has begun to question the truth of a sentence which held for centuries a conspicuous place in its proverbial philosophy. Mankind has so long acquiesced in the great truth that all men are mortal, that the majority have thought it unnecessary to inquire whether all men are equally so. Whilst philosophers and political economists have found in the various questions relating to the increase of the species an ample and attractive field of investigation; whilst physicians have directed their inquiries to the nature and treatment of isolated disease, or at most have been compelled to a wider survey by the ravages of some far spreading epidemic, the great problems constantly worked out around them in the actual death-rate of the people have hitherto met with few observers; their facts have been allowed to pass unchallenged; and, in place of careful deduction, has been advanced little but groundless assumption or vague surmise.

Yet, if we allow ourselves to reflect on the information conveyed by a numerical statement of mortality in the various districts of a country of known population, provided such statement be deduced from the observation of a sufficiently extended period, it will not be long ere we become aware of its vast importance as a subject of inquiry. The rate of death in a district is not a mere statistical formula; it is the most delicate index of the physical well being of its inhabitants; it represents the commercial value, in the widest sense, of its men, women, and children; its revelations are not simply of the actual loss of human life, but of the waste of human intellect

* An Inquiry into the different Proportions of Death produced by certain Diseases in different Districts in England. By EDWARD HEADLAM GREENHOW, M.D. With an Introductory Report on the Preventibility of Certain Kinds of Premature Death. By JOHN SIMON, F.R.S. Blue Book. London: 1858.

and human strength produced by morbific influences, only a small portion of which can be fatal; and, if it teaches with certainty of the present, it prophesies with equal certainty of the future. Whilst, on the one hand, all allow that national intellect and national wealth, the social advancement and political power of a people, are in direct relation with well developed brains and vigorous muscles, with stalwart arms and brawny shoulders, with healthy lungs and good stomachs; on the other, it is indisputable that a high death-rate indicates an unhealthy population, and that such a population must of necessity procreate an enfeebled offspring, a race incapable of high aims and great deeds. An undue national mortality can only be the precursor of political decay and national fall. The death-rate of England is the true pulse of the nation, the slightest increase of which from the normal standard imperatively demands the attention of all "statists and lovers of their country."

These remarks, which we acknowledge are somewhat trite, have been forcibly suggested by the perusal of the paper by Dr. Headlam Greenhow, published under the authority of the General Board of Health, which embodies the results of his *Inquiry into the Different Proportions of Death produced by Certain Diseases in Different Districts in England;* together with the Introductory Report by Mr. Simon, *On the Preventability of Certain Kinds of Premature Death.* To some of the facts and deductions at which these gentlemen have arrived, we would now invite the attention of our readers, premising that we do not think it easy to overestimate the value of Dr. Greenhow's labours, whether considered as a statement of truth, eliminated and worked out with much care and precision, or as pioneering the way to a most important and hitherto neglected field of scientific observation.

To identify the diseases which cause the surplus mortality in unhealthy districts, and to inquire what are the local and special morbific influences at work, originating or intensifying such dominant diseases, are the objects of Dr. Greenhow's labour. In the preparation of a course of lectures on Public Health, he found himself at a loss for materials in the shape of anything like exact information on these points. The principal sanitary literature of the day had dealt more in generalisations, frequently made from insufficient data, than in accurate deductions from ascertained facts. Because in many urban populations the mortality is far in excess of that which obtains in rural districts, it was hastily inferred that the great cause of preventable death throughout the country, was that neglect of well known sanitary laws which is especially incident to crowded communities. The prominence and magnitude of the evils accompanying or dependent on the compression of population within a limited area, overshadowed and obscured other causes of death and decay, which not less certainly, though less obtrusively, shed their fatal influences on the destinies of the people. Accordingly, with but few exceptions, our current teaching on public hygiene might be summed up under the three heads—ventilation, domestic and urban cleansing, and water-supply; and to enforce or improve these are the remedies which have been empirically recommended in all cases of more than average mortality. We would not for a moment be supposed to underrate the vast importance, as well as pressing necessity, of urging these matters on the national attention; we shall hereafter illustrate the considerable part their neglect plays in the causation of disease; but that reformation in these particulars is far from representing all that must be done ere England stands foremost in health and happiness, as she does in wealth and science, the facts which we shall submit to our readers will abundantly prove.

Again, there has prevailed hitherto considerable ignorance, at least in non-professional circles, as to the exact maladies which unnecessarily swell our death-roll. That the people are carried off by fever, cholera, and the other diseases usually included in the class zymotic, and that their agency is to a great extent controllable, most well informed persons are aware; but beyond this their knowledge does not extend. In fact, as Dr. Greenhow observes, the appellations zymotic and preventable are often used as convertible terms. To how many will the information be new, that pulmonary affections, including phthisis, cause nearly a quarter of our annual mortality; that phthisis alone kills every year more than fifty thousand persons; and that, of all our non-contagious diseases, it and its allied forms of death are most under the control of the living!

In the prosecution of his undertaking, Dr. Greenhow selected from the six hundred and twenty-three districts into which England and Wales are divided for the purposes of registration, a series of one hundred and five, comprising "a variety of healthy and unhealthy places, each of them distinguished by its position, character, or some peculiarity in the industrial employment of its inhabitants." The diseases whose influence he determined to estimate were, besides those of the zymotic class, those which arise from perverted or imperfect nutrition, including tubercular affections, together with disorders of the respiratory organs, convulsions, teething, apoplexy, paralysis, rheumatism, carbuncle, and phlegmon. A period of seven years, 1848-54, is the time over which his investigation extends; a duration which he believes to be sufficiently long to obviate any miscalculation from the accidental fluctuations of particular seasons. The occurrence of the Census in 1851 determined the selection of this particular series of years, the returns of population then made being employed as the divisors for calculating their death-rates. The following is the mode by which he arrived at his results :—

"The number of deaths occasioned in each district during the septennial period by each of the several diseases having been added together separately for each sex, the annual average death-rate of each sex from each disease has been carefully calculated, the population of 1851 being employed as the divisor. In order to afford a correct comparison between the several districts, it was necessary to bring the death-rates to one common denomination. To avoid the use of fractions, the death-rates have been calculated in each district for 100,000 persons; for, although it is true that very few districts contain 100,000 persons of each sex, and the entire population of most districts falls far short of 100,000, there is no real objection to the adoption of any standard for a comparison of the present kind, provided only the correct proportions be preserved."

The averages of mortality thus obtained have been compared with the death-loss from the same diseases in England and Wales taken as a whole, with that of each of the great registration provinces into which the country is divided, and with that of particular counties. Information is also appended, in the tables which form the groundwork of the paper, as to the number of inhabitants in each district; the average number of persons residing on each square mile; the number of persons in each hundred of the entire population, who dwell in towns; the number of paupers to each thousand inhabitants; together with the names of the most prevalent industrial occupations.

Supplemental to the major investigation which is above sketched out, twenty of the selected districts have been made the basis of a minute inquiry, which throws considerable light on the influence of age in determining the fatality of particular diseases. In each district have been computed "the average annual proportion of deaths per 100,000 children of each sex, below the age of five years, from all causes, and from each of the several diseases, phthisis, diarrhœa, and diseases of the respiratory organs"; "the average annual proportion of deaths from phthisis and from diseases of the respiratory organs, in adults above twenty years of age, per 100,000 of each sex"; and "the average annual proportion of deaths from typhus, in young persons under twenty years of age, and in adults above twenty years of age, per 100,000 of each sex."

Such being the character and scope of the work before us, before detailing any of the results at which the author has arrived, we may refer to a question which naturally suggests itself on the threshold of any such investigation; it is, What is the ratio of non-preventable death; in what proportion must the population of this country necessarily, year by year, succumb to the common fate of humanity?

This question has received special attention from the Medical Officer of the Board of Health, in his *Introductory Report;* and from his conclusions we shall draw our reply. Death from old age, from a physiological point of view the only natural mode of death, would, it may be believed, take place in most individuals about the age of eighty. In this sense, any death occurring before that period must be regarded as premature. Did all thus live out the full measure of their days, the annual mortality would amount to 1250 in every 100,000. But all premature death is not preventable. There are causes which nip infancy in its bud, and destroy manhood with "its blushing honours thick upon it", over which science, in its present state of advancement, exercises but a limited control. Such are congenital conditions and hereditary tendencies, the current contagions of hooping-cough, measles, and scarlatina, poverty and its concomitant evils, accidental injuries, criminal violence, and the long train of maladies which follow in the steps of vice and intemperance. Some of these are in the nature of things inevitable, others will only admit of mitigation to a partial extent, and by degrees; but to the question, What is the necessary augmentation of mortality resulting from the operation of these causes in the present state of society? at least an approximate answer is to be found in the varying death-rates of different districts.

In the ever restless and ever mingling population of such a country as England, where the intermixture of families is restrained by no class restrictions, where the facilities of human intercourse are greater than in any other, it cannot be affirmed that these causes are likely to have more than a temporary preponderance in one locality than another. Now in sixty-four districts, containing a million of inhabitants, the annual average mortality does not exceed from 1500 to 1700 in the 100,000; in other words, from 250 to 450 in excess of what it would be did the population reach the extreme verge of human existence. In assigning this as the highest range to which the death-rate from non-preventable causes should rise, we may rest assured we are not fixing too low a standard. Although it may be urged that many of these districts are rural, and therefore to a certain extent more removed from some of the fatal influences we have mentioned, yet, on the other hand, such immunity can be but temporary, and it must be counterbalanced by the large proportion which, in consequence of the constant migration of youth and adult life to the towns, infancy and old age preserve in our rustic populations. Wherever, then, the mortality exceeds the above ratio, the just conclusion is, that it depends on causes which are local, and, if local, certainly preventable; at least, the science of the nineteenth century would indignantly repel the charge of being powerless to their removal. Now the deplorable truth is that, in nine-tenths of the kingdom, the death-rate is in excess of the highest limit we have stated, viz. 1700 in the 100,000; the average of deaths for the whole country being 2,266, whilst in some districts it reaches the enormous amounts of 3,100, 3,300, and 3,600. By this calculation, then, we are inevitably led to the conclusion arrived at by the Registrar-General, that one out of every four persons who annually die in England falls a victim to the neglect of physiological laws; or, in other words, that 100,000 are each year cut off by causes of artificial production.

In endeavouring to elucidate the origin of this waste of human life, the natural mode of proceeding would appear to be to inquire what is the actual death-loss from diseases which are known to be more or less dependent on alterable causes; to observe in what localities these diseases exercise their fullest influence, and what are the peculiarities in the condition, occupation, or mode of living of the inhabitants, which raise them to such an unenviable preeminence. That cholera, dysentery, and diarrhœa, typhus and typhoid fevers, diseases of the respiratory organs, phthisis, and other strumous affections, the contagious diseases of childhood, and the nervous affections of infancy, are to be included in this category, is established on irrefragable evidence, a summary of which is to be found in Mr. Simon's *Report*. The total mortality from these diseases amounts to 227,000 per annum; the relative is as follows: the three diarrhœal diseases have, during the last eight or nine years, destroyed an annual average of 26,388; fevers, including typhus, typhoid, infantile, and remittent, 18,616; non-tubercular diseases of the respiratory organs, 50,273, of these deaths 23,020 have occurred during the period of childhood; tubercular disease, including phthisis, numbers its 57,982 victims; deaths by the infectious disorders, including small-pox, amount to 36,587; and 37,000 children are yearly swept away by those affections of the nervous system which find their chief development where infancy is "cribbed, cabined, and confined" in close rooms and ill ventilated wards, and in those centres of manufacturing industry, where, amid the din of the factory, the voice of maternal instinct is lost, and hecatombs of young lives are sacrificed to the necessities of the many and the emolument of the few.

Descending to particulars, and following the plan of the work before us, we shall treat these affections *seriatim;* and, commencing with the pulmonary class, which in Dr. Greenhow's arrangement is made to include phthisis, the first fact which attracts our notice is, that the inhabitants of agricultural districts enjoy a marked immunity from these diseases. The annual average mortality from all pulmonary affections is, in England and Wales, 569 for the male population, 535 for the female, in the 100,000. In two of the eleven great divisions of the kingdom, the pulmonary death-rate is greatly in excess; these are London and the north-western counties, the divisions which possess the largest urban population. In London the male death-rate is 758, and the female 593; in the north-western counties, the male 694, and the female 674. On the other hand, in purely agricultural districts, where there are no towns and the inhabitants are tillers of the soil, the annual average loss from these diseases varies from 216 (in Glendale) to 473 (New Forest) for each 100,000 of both sexes. Again, it is the large towns which exhibit the most excessive loss. Liverpool, the unhealthiest town in the kingdom, loses for every 100,000 of each sex 1062 males, and 939 females; Bristol, 979 males, and 742 females; and Manchester, 905 males, and 816 females. But we must pause ere we infer that the collection of human beings in urban communities is *necessarily* accompanied by so high a development of diseases of the breathing apparatus. There are many towns in England where pulmonary disease is under the general average. In Norwich, with a population exceeding that of Bristol (proper), the death-rate from this class of malady is 536; in Bedford, Bideford, Wellingborough, Knaresborough, Spalding, Whittlesey, Lewes, and Wisbeach, the death-rate is under 500 for both sexes; Whittlesey and Knaresborough are, in this respect, both healthier than the New Forest. Neither does a greater aggregation of the inhabitants within a given space necessarily give rise to an increase of this class of death. "The population density of Bristol", says Dr. Greenhow, "being considered as 100, that of Hull would be 77; of Gravesend, 30; and of Ipswich, only 11. Thus Hull has seven of its inhabitants crowded on the same space of ground that is occupied by a single individual in Ipswich. Yet Hull contrasts favourably with Ipswich as regards the proportion of deaths from pulmonary affections among its inhabitants; for Ipswich annually loses at the rate of 97 persons per 100,000 more from this class of diseases than Hull." In fact, the proportion in Hull is only three in excess of that of the entire kingdom. The conclusion we would draw is, that, although it cannot be denied that a certain predisposition to lung and bronchial disease is induced by urban residence, yet, on the other hand, the amount of such influence is *per se* comparatively limited; that it must be strengthened and enforced by other causes, ere it can suffice to poison the well-springs of life; and that, were such other sources of danger removed or lessened, the death-roll of our towns would never exhibit the disgraceful and deplorable results to which we have alluded.

To return, however, to the country; we should again err did we imagine that mere living apart from "tower'd cities and the busy hum of men", is in itself a passport to safety from pulmonary disease. It is in country districts, perhaps, that the full influence of occupation on the inhabitants is best recognised. Even purely agricultural pursuits are not equally healthful. A curious illustration of this seems furnished by the district of Hendon. The male death-rate from disease of the respiratory organs in Hendon is not only high in itself, but much higher than the female. The explanation offered is, that Hendon includes the great hay-growing district which lies between London and Harrow. Most of its agricultural labourers are employed as hay-binders or carters, and are thus constantly exposed to the inhalation of an irritating dust. Whether this be a correct explanation of the facts we cannot say, but at least it appears a curious coincidence. In a certain number of rural populations, the men are cultivators of the soil, whilst the women are engaged in various in-door manufactures. Of this the districts of Newport Pagnell, Bedford, Towcester, and Wycombe may be taken as examples. They are all lace-making districts; and in all of them the female mortality from lung affection is very considerably in advance of the male. The same rule prevails, although to a less extent, and liable to one or two exceptions, in localities where the women are employed in the manufacture of cotton, straw plait, and straw bonnets. How direct is the sequence between the occupation of lace making and the development of phthisis, is shewn most clearly by an examination of the death-rate of Towcester. In this district, the "excess of the death-rate from pulmonary affections" (principally phthisis) "in the adult women above the death-rate of men, is very nearly twice as high as the excess for the whole of life. Moreover, children perish in an undue proportion during the first years of life, both from all causes, and from affections of the chest; but the death-rate of male children from all causes, and from pulmonary diseases, is higher than the death-rate of female children by what must be considered as the normal amount."

Nowhere is the effect of occupation on health more clearly demonstrated than in the mining districts of England. In the pure bracing northern air of the romantic Cumberland uplands, in the mining districts of Wales, and on the rocky Cornish moors, where the soft western breeze is first met, wafted from the broad Atlantic—wheresoever the same industrial pursuit prevails, the same results are encountered. For several reasons, the calculations from which the fact of the injurious effect of metal-mining on the respiratory organs is deduced, are particularly free from sources of fallacy. Metalliferous mines are situated in most instances far away from the busy towns, in the midst of a rustic population unaffected by urban influences, and the people attracted to their vicinity are the comparatively small number who earn in them their sustenance, or minister to the wants of their workers. Few of the weaker sex are employed in them; in the whole of England, the class of women miners does not exceed in number eleven thousand The sanitary effects, therefore, of this department of labour, should be evinced with but little exception in the male population; the death-rate of the female affording an unimpeachable standard of comparison. Now the facts are these: the lead-mining district of Alston loses each year by pulmonary affections 877 males for every 494 females; in Reeth the proportion is 724 to 528; at Pately Bridge it is 508 to 391; and at Aberystwith 491 to 429. In the tin and copper mines of Cornwall, a similar comparison elicits similar results; in the district of Liskeard, the death-loss from chest-disease is, for the males 491, for the females 432; at Penzance, 560 for 456; and at Redruth, the comparative relation of the mortality in the two sexes is 670 to 450.

Whatever may be the effect of coal-mining in the production of carbonaceous deposit in the lungs, it would appear that, as far as these organs are concerned, this employment is incapable of giving rise to active disease: in Glendale 4 per cent. of the male population are coal-miners, in Easington 50 per cent.; yet the male death-rate of the former from pulmonary disease is 215, of the latter only 222 per 100,000. In coal-mining localities, also, the relative proportion of male and female mortality from affections of the breathing apparatus assumes what we must believe to be its normal condition, the female death-rate being a little higher than the male; but in parts of the country where mining operations are of a mixed character, as in the coal and iron districts of Wales and the North, the male mortality again rises in most instances above that of the other sex; and, although an undue proportion is not so marked as in the purely metalliferous districts, yet, as far as it goes, it bears us out in ascribing to metallic mining a direct influence in the production of pulmonary disease. Before leaving the subject of mines, we must advert to the fact that lead-mining is in advance of all other kindred occupations in originating this variety of morbific influence.

"The women of Liverpool", says Dr. Greenhow, "perish from chest-affections in a rather larger proportion than those of Reeth; in a slightly lower proportion than the women of Alston; but the men of Alston and Reeth die in a much larger proportion than the men of Liverpool. Thus a district remote from city influences, situated in the midst of a most salubrious district, and containing scarcely an appreciable urban character (since, although 29 per cent. of the inhabitants of Alston reside in the town that gives name to the district, the town only contained 2,005 inhabitants in 1851) loses a larger annual proportion of its adult male inhabitants from diseases of the chest, than the unhealthiest city in the kingdom. That this is due to the nature of the prevalent employment, no doubt can be entertained. It is the injurious character of the male occupation which causes Alston, the most exclusively lead-mining district in England, to be the place in which there is a larger proportion of widows than in any other place in the kingdom." (p. 63.)

There is another circumstance in connexion with this district, which may be quoted in illustration of a well known physical law, which we fear is obtaining elsewhere a far wider and more important theatre of operation. The law to which we allude is, that what was once an acquired peculiarity, becomes in process of time an hereditary predisposition, transmitted from generation to generation. Doubtless this is the true explanation of the high infantile and female pulmonary death-loss which Alston, for centuries a lead-mining district, sustains; a conclusion receiving countenance from the facts, that the inhabitants of lead-mining localities in the North are easily distinguishable from the coal-miners by a peculiarity of aspect, and that the chief adult male mortality is referrible to asthma, a disease the hereditary nature of which is becoming daily more justly appreciated.

From Alston and Merthyr Tydfil to Birmingham and Sheffield, from the Titans to the sons of Tubal Cain, is a natural transition. In the great seats of metal manufacture, we again find abundant evidence of the relation which subsists between occupation and pulmonary disease. Out of eleven districts distinguished by this species of industry, in ten the male deaths from chest-affections are more or less in advance of the female; in one only, Alcester, is the proportion reversed, and in this a large number of the men are engaged in agriculture, whilst the sexes are nearly equally employed in the special manufacture of the place, namely needle-making. As might be expected, the coarser kinds of metal work, such as iron-founding and nail-making, are not productive of the same amount of pernicious effect with some of the branches of cutlery manufacture, where the workmen inhale an atmosphere charged with fine mechanical particles. The pathology of the Sheffield grinder's disease forms part of the current medical knowledge of the day; we shall not, therefore, here do more than illustrate its effects by stating the male death-rate from lung-affections, as compared with the female, in the three districts, Ecclesall Bierlow, Birmingham, and Sheffield. In the first it is 571 for the female, 736 for the male; in the second, 699 for the female, 838 for the male; whilst in Sheffield, 839 of the latter sex die for every 670 of the former.

The other branches of industry which tend to augment the death-rate from pulmonary affections, are the manufactures of earthenware and textile fabrics, including woollen, silk, cotton, linen, flax, lace, and hosiery goods. These are all shewn by the tables before us to be more or less efficient in the causation of disease. In illustration of the agency of the latter class of occupation, a good example is offered by the mortality of the silk manufacturers of Macclesfield and Leek. These districts, "next to Coventry, are the two in which the largest proportion both of men and women are employed in the silk manufacture; they are, therefore, the districts in which we should expect most obviously to observe the influence of silk manufacture on health. In both places a considerable number of females are employed in the prevalent occupation, and in both places the female considerably exceeds the male death-rate. The excess for the whole of life is nearly equal in the two districts; but it is greatest in adult life among the women of Macclesfield, where a much larger number are engaged in this industrial employment. In Leek the pulmonary death-rate of adult women exceeds that of adult men in the proportion of 82 per 100,000. In Macclesfield the pulmonary death-rate of women exceeds that of men in the proportion of 148 per 100,000."

Thus far, we have been considering pulmonary diseases *en*

*masse;* and we have, we think, exemplified to a great extent the part played by occupation in their etiology. Affections of this class, however, divide themselves naturally into two great groups, the tubercular, and the non-tubercular; and which of these is most the product of artificial circumstances, may be a matter of dispute. Phthisis, a malady the predisposition to which is capable of transmission from parent to offspring with ever increasing force, is preeminently the disease of the crowded factory and close workshop. Dr. Baly has shewn how far phthisis is originated and fostered by the exclusion from sun and air, the absence of healthy mental stimulus, the monotonous employment, and the withdrawal from all the life-giving influences of external nature, which are the concomitants of penal confinement. Mr. Simon directs our attention to the analogy between life in the prison-house and life in the textile factory ; and that such is no chimera of a mere poetical philanthropy, is by the high phthisical death-rate in the great seats of manufacturing industry placed beyond reasonable doubt. A consideration of this fact becomes absolutely appalling, when we reflect that each adult in whom phthisis is thus originated, may, before he or she succumb to its power, transmit the tendency to children, who, remaining within the fatal circle, add again and again new victims to its deadly influences in an ever multiplying scale, or, floated away in the tide of migratory population, carry with them the germs of destruction to taint fresh springs of existence, and decimate new fields of industry.

That such results are the fated adjuncts of human civilisation, we will not believe. If hygienic teaching be not a fiction, if physiological laws be not the mere day-dreams of a speculative philosophy, civilised man should be the highest type of man, not in intellect alone, but in health in its fullest sense, in bodily vigour, in immunity from disease, and in the power of resisting its influences. This is the end which sanitary science proposes to herself; and to this goal she is capable of conducting any community that will obey her dictates. It is with pleasure we find that attention is already being directed by some of the responsible employers of national industry to the means whereby may be secured to their operatives the development of a well balanced mental in a healthy corporeal existence. From a paper by Mr. Akroyd, in the *Transactions* of the Social Science Association, Mr. Simon extracts the following description of the resources and inducements for healthy recreation, which the author has provided for his nearly five thousand work-people :—

"A library is attached to the works, to which any of my work-people have access free of charge. A news-room is provided, supplied with the newspapers of the metropolis and the locality, and also with the current periodical literature. A band is established at the works, and its performances are very creditable. It plays out of doors occasionally, when the weather is favourable; at other times, in a room provided for the purpose. Allotment gardens are provided for the workmen; and, in connexion therewith, a horticultural and floral society has been established, to promote the knowledge and cultivation of fruits, flowers, plants, and vegetables. An exhibition is held annually, at which prizes are given for the best productions of the respective gardens. To strengthen the habit of observation, and to cherish a taste for the beauties of nature, I give prizes for the best collection of wild plants and ferns growing in the neighbourhood. Recreation grounds are provided for the juvenile and adult members of the establishment, and every encouragement is given to the practice of healthy out-door sports and athletic games."

Is it possible to compute the benefits which would result, were the same means of health, moral training, and intellectual advancement, provided for all the swarming denizens of our manufacturing hives? Were such the case, we should be curious to know what amount in actual money would be annually saved in the one item of widows and orphans provided for at the public cost.

We have dwelt thus long on the pulmonary death-rate, because we conceive that the facts which it brings to light are not as generally known, and have not been urged on public attention in an equal proportion with some other truths of the same nature. Realities of not inferior moment are taught by the district mortality of the remaining wholly or partially preventable diseases which were named in the outset. Space, however, will not allow us to do more than cursorily glance at some of the more obvious of them.

Perhaps the most valuable boon which science has ever presented to mankind, was the discovery of vaccination. By it, amongst many civilised communities, the mortality from small-pox, which previously had numbered its victims by tens of thousands, has been reduced to an insignificant fraction; and it is difficult to conceive a reason why small-pox should not, as far as Europe is concerned, have already become as much a spectre of the past as the black death or sweating sickness. But the facts are that, in considerable districts of England, the land of Jenner, and sixty years after his discovery, during the three months ending March 31st, deaths by small-pox were amounting to a fourth part of the entire local mortality. In the kingdom, taken as a whole, the death-rate averages between four and five thousand annually; and, be it remarked, that this disgraceful waste of human life does not rest chiefly the opprobrium of rural districts, whose darkness is but partially dispelled by the illumination of science, but its amount is equally furnished by the statistics of our great towns. Plymouth and East Stonehouse have lost annually, during the last seven years, at the rate of 138 in the 100,000 inhabitants; Portsea, at the rate of 101; Derby, at the rate of 76; and Bristol, at the rate of 71. On the other hand, the mortality from small-pox during the same time in Bedford, only averaged 6 in the 100,000; in Stroud, only 8; and in Spalding and Lewes, only 4 each; whilst the districts of Reeth, Blofield, and Romney Marsh, lost by it none of their inhabitants. We are fully aware that, in consequence of the tendency which small-pox exhibits to epidemic prevalence, a low death-rate from that disease, unless the calculation be made from the returns of a very long period, does not convey completely unexceptionable evidence. It may have been that, during the selected period, the peculiar epidemic constitution was wanting, which, had it been present, would have considerably augmented its fatality. But, as is observed by Mr. Simon, the import of a high small-pox death-rate admits of no uncertain construction. It is to be taken as direct evidence, that the practice of vaccination is more or less systematically neglected. Viewed in this light, the small-pox returns published by the Registrar-General are a blot on the civilisation of the English people.

In consequence of the epidemic tendency which distinguishes the prevalence of measles, hooping-cough, and scarlatina, we will attempt to draw no inferences based on their district mortality. But the significance of their death-rate, as calculated for large masses of population, does not allow of misconception. We cannot mistake the meaning of the fact, that the two millions and a half who occupy the north-western counties, sustain yearly a loss by these diseases double in proportion to that inflicted on the four millions inhabiting the south-eastern quarter. Admitting the influence of a dense population in facilitating the transmission of their specific poisons, and the early age at which, in consequence of such rapid transmission, individuals are exposed to their infection, still the immense preponderance of their fatality is surely a proof that other causes are at work to augment their virulence and swell the number of their victims. And this conclusion acquires a tenfold force from the fact that the non-contagious maladies of childhood are equally dominant in these counties. Taken together, they afford sure ground for the deduction that, in the great centres of English industry, influences prevail which on the one hand intensify the malignity of contagious disease, and on the other, by undermining the energies of infant vitality, prostrate its conservative power.

Alvine flux, under which term Dr. Greenhow includes the three diseases cholera, diarrhœa, and dysentery, has caused during the nine years, 1848-56, 237,498 deaths. This prodigious mortality has of course been distributed very unequally over the period. In the two cholera years, 1849 and 1854, "there were 116,246 deaths; in the two years 1850 and 1855, there were but 29,425, or little more than a fourth part of the former amount." Now the great fact which is brought to light by the statistics of these diseases, is the "astounding inequality" which has marked their local prevalence. Taken as a whole, there can be no doubt that they are increasing amongst us; but their death-rate varies in different districts from nothing, or next to nothing, to between six and seven hundred in the 100,000 inhabitants. Liverpool and Hull are examples of the highest mortality, where the death-rate reaches 662 and 559 respectively; Aberystwith of the lowest, where the death-rate from the three diseases is represented by an annual average of 4 in the 100,000. And be it observed that this inequality does not merely apply to the eminently "fitful and erratic" disease cholera, but a similar disproportion is observed in those diarrhœal affections which may be considered indigenous to our soil. Dr. Greenhow, indeed, shews that cholera itself has never been absent from some of our great towns during the period over which his observation extends, and that in some localities, in 1852, it prevailed epidemically; but the death-rate from ordinary diarrhœa and dysentery varies from less than 10 in some districts, to 305 and 345 in others. And instances of places are not wanting, which have to a considerable extent escaped the visitation of cholera, but have suffered a high death-loss from diarrhœa. Now, whatever may be the exact process of causation in cholera, it is admitted on all hands that these diseases are the immediate result of the introduction into the system of organic impurities.

"Nothing in medicine is more certain", says Mr. Simon, "than the general meaning of high diarrhœal death-rates. The mucous membrane of the intestinal canal is the excreting surface to which nature directs all the accidental putridities which enter us. Whether they have been breathed, or drunk, or eaten, or sucked up into the blood from the surface of foul sores, or directly injected into blood-vessels by the physiological experimenter, there it is that they settle and act. As wine gets into the head, so these agents get into the bowels. There, as their universal result, they tend to produce diarrhœa; simple diarrhœa in the absence of specific infections; specific diarrhœa when the ferments of cholera and typhoid fever are in operation."

If this be the case—and to the general doctrine we cannot refuse our adherence—how completely must this class of disease be under the control of society; and what startling proportions does this consideration assume, when we are informed that, "if the diarrhœal death-rate of England generally were *ten times* the minimum local diarrhœal death-rate, there would be an annual saving in England of nearly 20,000 lives!"

Did our limits permit, we might draw from the statistics of continued fever facts equally pregnant. We can only record that 17,371 persons are each year sacrificed in this country to typhus and typhoid fever, forms of death which the experiences of every camp, hospital, and gaol, in modern Europe have proved to depend, either for origination or development, solely on causes of human production.

Before we conclude, however, we would especially draw the attention of the reader to the facts connected with infantile mortality, and in particular to that arising from the nervous diseases of childhood. There die annually of the three affections described in the Registrar's report as convulsions, hydrocephalus, and teething, 37,000 children; taken together with the other principal non-contagious diseases of young life, viz. diarrhœa and pulmonary inflammations, these year by year conjointly destroy about 72,000, and constitute one-sixth of the entire mortality of England. Now the district mortality from the convulsive disorders of childhood varies in different localities from 280 to 3,832; from infantile pulmonary affections, from 213 to 2,897; from infantile diarrhœa, from 28 to 1779; and it is, as a rule,* in the great industrial urban districts that the chief excess of mortality obtains, in fact, under the same local circumstances which augment the death-rate from measles, scarlatina, and hooping-cough. That the convulsive diseases of infancy are produced and fostered by a foul vitiated atmosphere, is placed beyond question by the experience of the Dublin Lying-in Hospital, where, by ventilation of the wards, the mortality from convulsive affections of new-born children was reduced to one sixty-

* The three Welsh districts, Carnarvon, Merthyr Tydfil, and Wrexham, present a very high mortality from the nervous diseases of childhood. The remarkable excess which in this particular characterises Carnarvon, an otherwise healthy district, must be considered exceptional, although at present inexplicable.

eighth of what it had been fifty years previously. With such a fact before us, can we reasonably doubt that the admission of fresh air into the close rooms and unventilated lodging-houses of our working population would be a step, and a considerable one, towards saving some of the 37,000 children annually sacrificed? Another point to be observed is, that it is in the factory districts where female labour is most in request, where children are deprived of nourishment from the mother's bosom and lack exercise in the mother's arms, that the human blossom most surely falls unexpanded. And, remembering that a high infantile death-rate is not merely the standard of mortality, but is the gauge of health and strength in the young population, that for every infant that dies another struggles through the same sickly childhood, to emerge a stunted feeble being, incapable of high physical or psychical development, we cannot shut our eyes to the terrible truth implied in the deduction, "that high local mortalities of children must almost necessarily denote a high local prevalence of those causes which determine a degeneration of race."

---

